# Differential patient selection for amyloid‐targeting immunotherapies in Alzheimer’s disease

**DOI:** 10.1002/alz.091478

**Published:** 2025-01-03

**Authors:** Jurij Rosen, Frank Jessen

**Affiliations:** ^1^ Medical Faculty, University of Cologne, Cologne Germany; ^2^ German Center for Neurodegenerative Diseases (DZNE), Bonn Germany; ^3^ Excellence Cluster on Cellular Stress Responses in Aging‐Associated Diseases (CECAD), Faculty of Medicine and University Hospital Cologne, Cologne Germany; ^4^ Department of Psychiatry, University of Cologne, Medical Faculty, Cologne Germany

## Abstract

**Background:**

Anti‐amyloid beta (Aβ) immunotherapies have recently evolved as promising treatment options in patients with early symptomatic Alzheimer’s disease (AD). Meanwhile, accurate patient selection for these treatments might be challenging mirrored by strict selection criteria of the hitherto published clinical trials. Based on a real‐world tertiary care sample of post‐mortem validated cases, we aimed to identify the patients matching these selection criteria and their proportion showing post‐mortem AD pathology.

**Method:**

This study is based on data from The National Alzheimer‘s Coordinating Center‐Uniform and Neuropathology Data Set. Patients with available post‐mortem report of AD pathology were selected by the published selection criteria of clinical trials evaluating efficacy and safety of aducanumab, lecanemab and donanemab. The resulting samples were scrutinized for a potential overlap and their proportion showing post‐mortem AD pathology.

**Result:**

Of 44359 patients in the dataset, 3343 had an available report of post‐mortem AD pathology. Of these, 887 patients were potential candidates for an anti‐Aβ immunotherapy as they presented an amnestic AD syndrome at the baseline visit. Applying selection criteria of clinical trials on these patients resulted in 91, 306, and 192 patients that would be suitable for immunotherapy with aducanumab, lecanemab and donanemab, respectively. These patient samples showed a partial overlap. In these samples, 83, 288, and 184 patients showed post‐mortem AD pathology.

**Conclusion:**

The present results might influence both differential patient selection for upcoming anti‐Aβ immunotherapies and interpretability of potential differences of clinical trial results.

